# Cabazitaxel-Induced Stabilization of Microtubules Enhances Radiosensitivity in Ovarian Cancer Cells

**DOI:** 10.3389/fonc.2013.00226

**Published:** 2013-09-18

**Authors:** Charles A. Kunos, Tammy Stefan, James W. Jacobberger

**Affiliations:** ^1^Department of Radiation Oncology, Case Western Reserve School of Medicine, Cleveland, OH, USA; ^2^Department of General Medicine, Case Western Reserve School of Medicine, Cleveland, OH, USA

**Keywords:** cabazitaxel, Jevtana, ovarian cancer, radiation

## Abstract

**Background:** Up to 40% of women with ovarian cancer have short disease-free intervals due to molecular mechanisms of chemotherapy resistance. New therapeutic strategies are sought. Ovarian cancers are sensitive to radiochemotherapy. The taxane cabazitaxel (XRP6258, Jevtana) promotes tubulin assembly and stabilizes microtubules against depolymerization in cells, acting similarly in mechanism to paclitaxel. Here, sequences of cabazitaxel-radiation co-administration are tested for drug-alone cytotoxicity and optimal radiosensitization.

**Materials and Methods:** SKOV3, OVCAR3, and TOV-112D ovarian cancer cells were administered cabazitaxel 24 h before (first), 18 h before (second), together (third), or 24 h after (fourth) a single radiation dose, and then, investigated by clonogenic assay and flow cytometric assays. Radiation dose-cell survival data were fitted by two-stage multivariate analyses of variance. High-content flow cytometry partitioned cabazitaxel effects into G2-phase versus M-phase events by DNA content, cyclin A2, and phospho-S10-histone H3 (PHH3). Paclitaxel served as a comparator.

**Findings:** Cabazitaxel cytotoxicity and radiosensitization were dose dependent. Cabazitaxel added 24 h before radiation was the most lethal schedule. DNA content measurements by flow cytometry showed that cabazitaxel-treated cells accumulated in the radiosensitive G2/M 4C DNA complement compartment. Cytometry also showed that surviving cabazitaxel-induced cell cycle arrested cells resolve the arrest by entering 4C or by 8C DNA complement cell cycles.

**Interpretation:** The radiosensitizing effect of cabazitaxel was schedule dependent, due to cell cycle redistribution, and best when cabazitaxel was given 24 h before radiation. Clinical trials of administering both cabazitaxel and radiation should be explored in women with chemoresistant ovarian cancer.

## Introduction

Despite objective response rates of 60–80% to platinum-based chemotherapy integrated with maximum cytoreductive surgery (1), the majority of the 22,240 women diagnosed with advanced-stage ovarian cancer in the United States in 2013 ultimately will develop progressive disease and will die of complications of their cancer. Up to 40% of these women will have a short cancer-free interval due to platinum-refractory disease recurring within 6 months (2). For platinum-refractory disease, a radiation-taxane therapeutic strategy may be appealing, stemming from experience gained in prior phase I clinical trials (3).

Assembly of chromosomes during the G2/M-phase of the cell cycle leaves cells not only vulnerable to death-provoking DNA double-strand breaks from ionizing radiation (4, 5), but also renders them sensitive to “poisons” of the mitotic spindle (6, 7). The taxane paclitaxel and its synthetic analog docetaxel block G2/M-phase transition by promoting and stabilizing tubulin polymers. While taxanes have lethal cell effects on their own (8), supraadditive cytotoxic effects are generally found when taxanes precede ionizing radiation (9–12). Radiation-taxane additive effects are observed when cells are exposed to brief pre-irradiation (13) or post-irradiation taxane incubations (14). Nevertheless, controversy exists. Both supraadditive effects (9, 10) and subadditive effects (15, 16) are noted when irradiated cells are incubated for ≥24 h in taxane-containing media. Mechanisms of untimely radiation-induced G1-phase block and M-phase cell cycle arrest have been put forward as means by which cells may escape taxane-related cytotoxicity (15, 16). Because of the mixed radioresponsiveness of cancer cells to radiation-taxane combinations, putative novel taxane and radiation combinations must be subject to extensive molecular *in vitro* investigations prior to clinical trial implementation.

Clinically, women having ovarian cancers that relapse after platinum and paclitaxel-based chemotherapies have therapeutic responses to an 8 Gy × 3 fraction stereotactic ablative radiosurgery (SABR) (17). However, disease progression may occur beyond the dosimetric contours of SABR-targeted disease. This brings to attention the need for a chemotherapeutics with radiosensitizing and outright cytotoxic properties that could be safely combined with SABR. The novel taxane cabazitaxel (XRP6258, Jevtana) promotes tubulin assembly and stabilizes microtubules against depolymerization in cells, acting similarly in mechanism to paclitaxel (18). Cabazitaxel was selected based on its pre-clinical activity in cancer cells known to be resistant to taxanes, a proof-of-concept achieved in clinical studies (19, 20). Pre-clinical data for cabazitaxel have identified an IC_50_ ranging 0.003–0.029 μmol/L, a 1-h post-infusion peak concentration followed by 6 h therapeutic range in humans, and triphasic elimination of the drug such that it possesses a long terminal half life (20, 21). Utilizing these pharmacokinetic parameters, we specifically tested the hypothesis that cabazitaxel enhances radiation-related cell lethality by inducing G2/M-phase cell cycle accumulation prior to radiation exposure.

## Materials and Methods

### Cell cultures and chemicals

Human ovarian cancer cells OVCAR3 [P-glycoprotein multiple drug resistance transporter 1 (*mdr-1*) positive, p53-mut (codon 248) (22)], SKOV3 [*mdr-1* positive, p53-mut (codon 179) (22)], and TOV-112D [P-glycoprotein multiple drug resistance transporter 1 (*mdr-1*) negative and p53-mut (codon 175) (23)] were obtained from American Type Culture Collection (Rockville, MD, USA). A *mdr-1* transporter may allow cells to evade taxane cytotoxicity (24). Cultured cells were maintained at 37°C in a humidified 5% CO_2_ atmosphere using Eagle’s minimum essential medium (Grand Island, NY, USA), with 10% fetal bovine serum, 1% non-essential amino acids, and 1% penicillin/streptomycin added. Cells were plated for 24 h prior to any radiation or drug exposure to generate exponentially growing cell populations. Chemicals used were purchased from Sigma (St. Louis, MO, USA) unless otherwise stated.

### Radiation and drug treatments

Radiation was delivered using a ^137^Cs γ-irradiator (JL Shepherd Associates, San Fernando, CA, USA) at 325 cGy per minute. Cabazitaxel (Jevtana, XRP6258) was an investigational agent provided to Case Western Reserve University (Cleveland, OH, USA) under an agreement with Sanofi-Aventis (Bridgewater, NJ, USA). To interfere with mitotic spindle activity (18), cabazitaxel was used at end concentrations of 0–10 μM (25). As a comparator, commercially available paclitaxel was used as indicated at the clinically relevant end concentrations of 0–10 μM (26). For assays with cell harvest times greater than 6 h, drug-containing medium was exchanged for drug-free medium 6 h after the start of the drug.

### Cell viability assays

Triplicate replicates of 1 × 10^4^ OVCAR3, SKOV3, and TOV-112D cells were incubated in 96-well plates for each indicated cabazitaxel or paclitaxel dose. Cells either underwent sham irradiation or a conventional clinical radiation dose (2 Gy) at the start of cabazitaxel or paclitaxel exposure. Six hours after the indicated treatment, exchanges for drug-free medium were done. After 18 h (i.e., 24 h after the start of drug exposure), cells were re-incubated in 300 μL of drug-free medium plus MTT (3-(4,5-Dimethylthiazol-2-yl)-2,5-diphenyltetrazolium bromide, 5 mg/mL). Following 3 h of incubation at 37°C, 96-well plates were analyzed by a spectrophotometer (Perkin Elmer, Waltham, MA, USA) for quantified colorimetric absorbance at an excitation wavelength of 540 nm. Means and standard errors (SE) reflecting viable cell number were plotted graphically. Analyses of fitted dose-response curves determined the amount of drug (IC_50_) that reduced viable cell number by 50% of control cells (27).

### Clonogenic survival assays

Exponentially growing OVCAR3, SKOV3, and TOV-112D cells were plated in triplicate on 24-well dishes to yield 300 cells (0, 2, 4, 8 Gy) or 3000 cells (20 Gy) per well. Cells underwent radiation or radiation with 6 h cabazitaxel (1 μM), an end concentration guided by human pharmacokinetic data (20). Four different radiation-cabazitaxel treatments were administered – cabazitaxel given 24 h before (24 h+, first schedule), or 18 h before (18 h+, second schedule), or together (0 h, third schedule), or 24 h after (24 h−, fourth schedule) a single radiation dose (2 or 8 Gy). Surviving colonies (>50 cells) were stained with 0.1% crystal violet in 70% ethanol 14 days after plating. Untreated cell cloning efficiency was normalized to 100%. Cloning efficiency of treated cells was expressed as a proportion of treated control survival. Two-stage multivariate analyses of variance (MANOVA) statistics analyzed radiation-cabazitaxel interactions (28, 29). MANOVA statistics (α = 0.05) were computed using statistical software (SPSS 18.0, Chicago, IL, USA). A survival fraction (SF) ratio of combined radiation-cabazitaxel treatment (Src) relative to the product of survival after radiation (Sr) and after cabazitaxel alone (Sc) determined additivity status. A Src/(Sr × Sc) ratio less than 1 indicates a supraadditive interaction; a Src/(Sr × Sc) ratio greater than 1 designates subadditive interaction.

### High-content flow cytometry for cell cycle analysis

**Figure A1:**
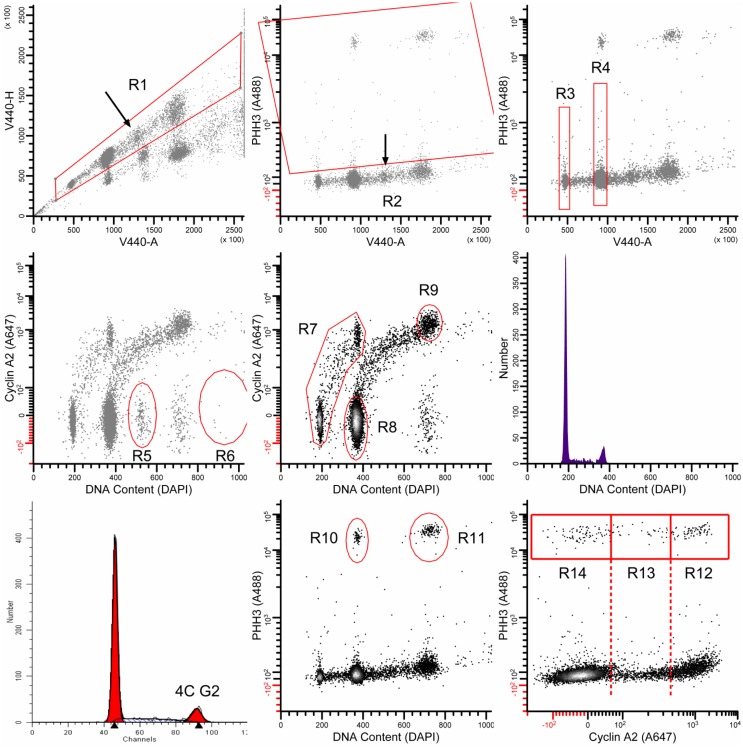
**The multi-parametric data are analyzed as follows: the region R1 gates in singlet cells and excludes all else; the region R2 classifies all mitotic cells**. The regions R3 and R4 were used to determine the median value of integrated DAPI fluorescence signal from the violet laser (V440-A). These values were used to transform DAPI fluorescence measurements of G1 and G2/M cells to mean values of 180 and 360, respectively [labeled DNA Content (DAPI)]. R5 and R6 were used in some samples to remove additional doubles that were missed by the doublet discriminator (arrow in upper left panel). These are recognizable by ∼6C content, G1 levels of cyclin A2 signal, and a distinct cluster. R7 was used to isolate the interphase stem line (2C → 4C). R8 and R9 were used to quantify 4C G1 and 8C G2 cells, respectively. The middle, right panel shows the DNA content histogram for the stem line, gated on (R1 AND R7 AND NOT R2). This histogram was analyzed by ModFit LT 3.2 (Verity Software House) to quantify 4C G2 cells (bottom, left panel). R10 and R11 were used to quantify the bulk of mitotic cells. These gates exclude early prophase and late telophase/cytokinetic cells. Finally, R12, R13, and R14 were used to quantify cells in the P4, PM, and M-LM mitotic states. For further description of mitotic states, see Ref. (16, 17).

Exponentially growing TOV-112D and SKOV3 cells were plated on 100 mm dishes to yield 2.0 × 10^6^ colonies per dish. To study cell cycle-related molecular events at the G2/M-phase transition, cells underwent radiation (0 or 2 Gy) and/or 6 h 1 μM cabazitaxel treatment. Trypsinized cultures were fixed and stained for DNA content by propidium iodide (PI) for standard flow cytometry or 4,6-diamidino-2-phenylindole (DAPI) with fluorescent antibodies reactive with cyclin A2 and phospho-S10-histone H3 (PHH3) as described (30, 31). Here, we discuss 4C cells, which are cells with four genome complements. 4C cells can be stemline G2, or stemline M, or 4C G1 cells of endoreduplicated (or bi-nucleate) progeny. Measuring the levels of cyclin A2 and PHH3 allow mapping of G2 and M cells as well as early (prophase, prometaphase) and later (metaphase, anaphase) stages of mitosis. Cytometry analyses were performed on a Beckton Dickinson LSRII flow cytometer (San Jose, CA, USA) using violet, blue, and red excitation and standard filter set-up. For PI stained cells, fractions of G1-, S-, and G2/M-phase cells were calculated using ModFit LT (Verity Software House, Topsham, ME, USA). For multi-parameter cell cycle analyses, sequential region mapping in two parameter space was performed as before by our group (30) using WinList 7.0 (Verity). An analytic walk-through example is shown in Figure A1 in Appendix.

## Results

### Cabazitaxel impact upon ovarian cancer cell viability

**Figure 1:**
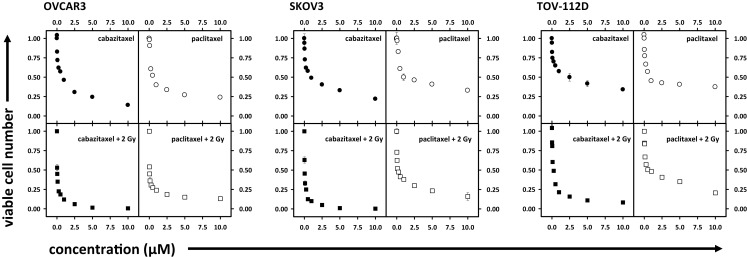
**Depicted are cell viability assays (MTT) in OVCAR3, SKOV3, and TOV-112D ovarian cancer cells**. Radiation (2 Gy), cabazitaxel, or paclitaxel treatments are indicated. Means and standard errors are plotted.

Ovarian cancer cell viability was determined by MTT assay. Figure 1 shows cell viability as a function of escalating concentration of cabazitaxel or of paclitaxel ranging from 0.025 to 10 μM. A 6 h exposure either of cabazitaxel or of paclitaxel minimally affected OVCAR3, SKOV3, or TOV-112D cell viability at the lowest concentrations of each drug (0.025–0.1 μM). In OVCAR3 cells, the IC_50_ was 0.6 μM for cabazitaxel and 0.7 μM for paclitaxel. In SKOV3 cells, the IC_50_ was 0.6 μM for cabazitaxel and 1.0 μM for paclitaxel. In TOV-112D cells, the IC_50_ was 0.6 μM for cabazitaxel and 0.6 μM for paclitaxel. The response to cabazitaxel and paclitaxel were similar among the three cell lines. As OVCAR3 and SKOV3 cells are both positive for *mdr-1*, any subtle differences in taxane sensitivity between the cell lines possibly may be related to an as yet untested differential affinity of the drugs to the *mdr-1* transporter. More study on *mdr-1* positive cells is warranted. TOV-112D cells lack an *mdr-1* transporter and because of this may be slightly more sensitive to taxane cytotoxicity. We do acknowledge that MTT cell metabolism viability assays do not distinguish among drug-related cell death or arrest of cell proliferation, both leading to an overall fewer number of cells capable of metabolizing the MTT introduced in the assay.

Figure 1 also shows ovarian cancer cell viability after drug exposure plus 2 Gy radiation, with this data normalized for 2 Gy radiation effect alone. Across all three cell lines, increase in cabazitaxel concentration substantially enhanced radiosensitivity. Two-step iterations comparing point-by-point cell viability and overall curve-fit indicated that radiosensitivity was enhanced more after cabazitaxel than after paclitaxel (*P* < 0.01 each, Figure 1). In OVCAR3 cells, the radiation-drug IC_50_ was 0.03 μM for cabazitaxel and 0.03 μM for paclitaxel. In SKOV3 cells, the radiation-drug IC_50_ was 0.04 μM for cabazitaxel and 0.11 μM for paclitaxel. In TOV-112D cells, the radiation-drug IC_50_ was 0.1 μM for cabazitaxel and 0.5 μM for paclitaxel. Looking at the IC_50_ responses of radiation-cabazitaxel treatment, subtle differences in sensitivity may be due either to our observed differences in cell doubling times (40, 36, and 30 h for OVCAR3, SKOV3, and TOV-112D, respectively) or to additivity effect.

### Cabazitaxel added before radiation maximizes ovarian cancer cell radiosensitivity

**Figure 2:**
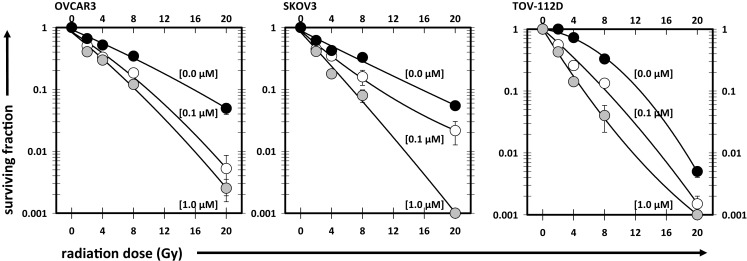
**Illustrated are clonogenic survival assays in OVCAR3, SKOV3, and TOV-112D ovarian cancer cells**. Cabazitaxel [black circles (0 μM), white circles (0.1 μM), gray circles (1 μM)] was added at the start of the assay for 6 h; drug-free medium was exchanged afterward for the duration of the assay. Mean and standard errors are presented. Fitted linear-quadratic regressions are plotted.

Cell survival after radiation or after radiation-cabazitaxel was established by clonogenic assays (Figure 2). Two escalated end concentrations of cabazitaxel (0.1, 1.0 μM) were selected for radiosensitization testing based upon our radiation-drug IC_50_ responses. In all three cell lines investigated by clonogenic survival, both concentrations of cabazitaxel-induced a significant decline in cell survival over a range of radiation doses (MANOVA *P* < 0.01 for each cabazitaxel concentration, compared to radiation alone). Detected differences in survival between radiation-cabazitaxel (0.1 μM) versus (1 μM) did not reach statistical significance in any cell line (MANOVA *P* > 0.08). Here, we were most interested in the magnitude of radiosensitization at the 8 Gy dose because this dose of radiation was of particular clinical interest to our SABR program (17). At an 8 Gy radiation dose given together with a 6-h 1 μM cabazitaxel exposure, a slightly more than additive interaction was detected. SF ratios were 0.98 for OVCAR3, 0.85 for SKOV3, and 0.99 for TOV-112D cells.

Our next step explored the effects of 1 μM cabazitaxel on the proportion of cells residing in G2- and M-phases of the cell cycle by cytometry. To mimic clinical pharmacokinetics of cabazitaxel, drug-containing media was exchanged for drug-free media at the 6 h time point after initial cabazitaxel exposure. We focused cytometry upon the initial 24 h post-exposure cell cycle kinetics period because were interested in testing putative, clinical workday feasible radiation-cabazitaxel schedules. Table 1 indicates that 1 μM cabazitaxel at least doubles the number of cells stalled at the G2/M-phase of the cell cycle 6 h post-exposure. The proportion of cells G2/M-phase-locked broadens over time and becomes maximal at the end of the first 24 h. Recovery studies showed slow cycling out of the G2/M-phase arrest and implicate resolution of the arrest by entering 4C or 8C DNA complement cell cycles (discussed below).

**Table 1:** **Redistributed G2/M cell cycle proportion after 6-h 1 ***μ***M cabazitaxel exposure (%)**.

Cell line	Hours after cabazitaxel^a^				
	0 h	6 h	12 h	18 h	24 h
OVCAR3	12	26	37	35	43
SKOV3	13	38	51	50	61
TOV-112D	8	33	47	46	58

^a^From three experiments; standard error <1%.

**Figure 3:**
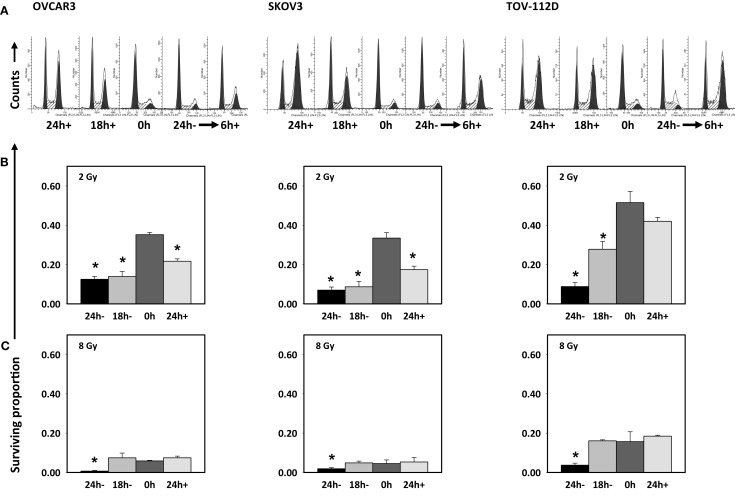
**Cell cycle analyses are depicted after 6 h 1 μM cabazitaxel exposure alone in OVCAR3, SKOV3, and TOV-112D ovarian cancer cells (A)**. Cabazitaxel was given 24 h before (24 h+, first schedule), 18 h before (18 h+, second schedule), together (0 h, third schedule), or 24 h after (24 h−, fourth schedule). Corresponding clonogenic survivals for 1 μM cabazitaxel preceding (24 or 18 h−), together (0 h) or after (24 h+) radiation are graphically presented for 2 Gy **(B)** or 8 Gy **(C)** irradiation. Means and standard errors are shown. Stars indicate significance at *P* < 0.01.

Then, four schedules of combined radiation-cabazitaxel treatment were investigated by cytometric assay and by clonogenic assay (Figure 3). Cabazitaxel was given 24 h before (24 h+, first schedule), 18 h before (18 h+, second schedule), together (0 h, third schedule), or 24 h after (24 h-, fourth schedule) a single radiation dose (2 or 8 Gy). The third (0 h) co-administered radiation and cabazitaxel schedule showed no perturbation in cell cycle progression by cytometry (Figure 3A), and treatment was essentially additive (SF range 0.85–1.25 for all cell lines). Therefore, the third schedule was arbitrarily assigned as the reference treatment by which other schedules would be compared for optimal radiosensitization.

The first schedule of 1 μM cabazitaxel given 24 h before radiation (24 h+) was supraadditive. Cell cycle analyses indicated that many cells had become stalled at the G2/M transition 24 h after cabazitaxel exposure (Figure 3A). 24 h+ cells were significantly sensitive to radiation (*P* < 0.01, compared to 0 h schedule, Figures 3B,C). SF ratios of 0.77, 0.20, and 0.17 at 2 Gy, and SF ratios of 0.10, 0.11, and 0.25 at 8 Gy were logged for OVCAR3, SKOV3, and TOV-112D cells, respectively.

The second schedule of cabazitaxel 1 μM given 18 h before radiation (18 h+) was also supraadditive. Cytometry indicated that 18 h after cabazitaxel a lower cell proportion had stopped cell cycle progression at the G2/M transition (Figure 3A). This could reflect two phenomena. First, cells originally in G1- or S-phase at the time of cabazitaxel exposure may have been suspended at the G2/M transition by this 18 h time point. Alternatively, cells initially in G2/M could have escaped the cell cycle block by cabazitaxel and now be captured by cytometry in a new G1- or S-phase state. Still, an 18 h+ schedule invoked high radiation-related cell kill in each cell line (Figures 3B,C). At 2 Gy, SF ratios were 0.83 for OVCAR3, 0.25 for SKOV3, and 0.53 for TOV-112D cells. At 8 Gy, SF ratios were 0.69 for OVCAR3, 0.27 for SKOV3, and 0.99 for TOV-112D cells. While OVCAR3 and SKOV3 cells retained supraadditive radiochemosensitivity at the two radiation doses, TOV-D cells showed less combined radiation-cabazitaxel responsiveness at 8 Gy. We put forward that this nearly additive interaction likely relates to a lack an *mdr-1* transporter in TOV-112D cells. Absence of an *mdr-1* transporter and therefore less efflux of the drug from the cell possibly enhances cabazitaxel-related cell kill, lowers an observed Sc, and raises SF. Given ablative radiation (8 Gy) has a substantial negative impact upon TOV-112D cell survival already, it is not unreasonable to observe a merely additive effect in these particular cells. On the other hand, data could support the contention that disrupted apoptosis signaling or differential drug to target affinity mitigate the effects here. Further testing is warranted.

The fourth schedule of radiation followed 24 h later by a 6-h exposure to cabazitaxel 1 μM (24 h−) resulted in mixed subadditive or additive interactions. For this schedule, cytometry detected only minimal perturbations in cell cycle proportions at the start of cabazitaxel exposure (Figure 3A). In lower cytometry channel numbers, PI-signal indicated that some cells had lost DNA and remained damaged at this 24 h post-irradiation time point. Within 6 h, cabazitaxel-induced a modest rise in G2/M block, with this effect most pronounced in the relatively quick cycling TOV-112D cells (30 h doubling time) as compared to OVCAR3 (40 h doubling time) and SKOV3 (36 h doubling time). Clonogenic assays revealed modest cytotoxicity attributable to a 24 h− schedule, but less so as compared to the first (24 h+) and second (18 h+) schedules. When assessing a combined 24 h− schedule for radiation-cabazitaxel interactions, it may be the case that death-provoking DNA damage unrepaired after radiation and lethal stabilization of mitotic spindles after cabazitaxel sequentially may not overlap to result in synergistic cytotoxicity. OVCAR3 cells showed a mixed subadditive SF ratio of 1.29 at 2 Gy but a more than additive SF ratio of 0.69 at 8 Gy. The mixed responsiveness to a radiation then cabazitaxel treatment n OVCAR3 cells may be due to a yet to be explained greater sensitivity to cabazitaxel alone (see also Figure 1). SKOV3 cells showed supraadditive SF ratios of 0.48 at 2 Gy and 0.27 at 8 Gy. TOV-112D cells exhibited a more than additive interaction at 2 Gy with a SF ratio of 0.79, but a subadditive interaction at 8 Gy with a SF ratio of 1.11. Mixed responsiveness in TOV-112D cells may be related to a greater sensitivity to ablative (≥8 Gy) radiation alone (see also Figure 2).

### Resolution of G2/M cell cycle arrest induced by cabazitaxel

Drug-induced biologic effects as a function of the cell cycle may be monitored by DNA content flow cytometry and readily distinguish cell proportions residing in the G1 and S phases of the cell cycle. But cells with four genome complements (4C cells) can exist in G2, M, or 4C G1 phases that results from either endoreduplication or failure to undergo cytokinesis, giving rise to a bi-nucleate cell cycle. To explore the effects of cabazitaxel at the G2/M transition, we employed multi-parameter analysis using immunodetection of DNA content, cyclin A2 as an indicator of early mitosis, and phospho-S10-histone H3 whose elevated expression during the decline of cyclin A2 maps late mitosis.

**Figure 4:**
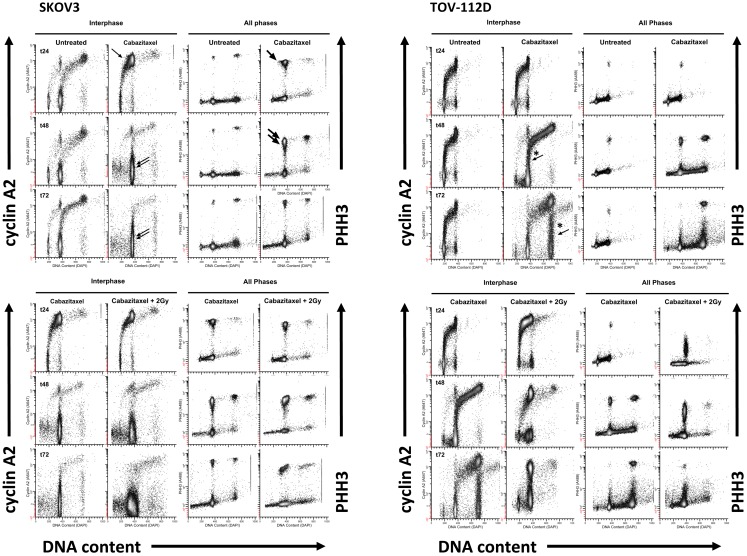
**High-content flow cytometry data are depicted for SKOV3 and TOV-112D cells**. A 6-h cabazitaxel (1 μM) exposure was studied; drug-free medium was exchanged at the 6-h time point. 24 h (t24), 48 h (t48), and 72 h (t72) after initial cabazitaxel treatment is indicated for each row of cytometry data. Arrows highlight clusters that represent accumulation or arrest at 4C G1 (thin arrow); 4C M (thick arrow, thick double arrow); 4C G1 (thin double arrow); 4C → 8C and 8C → 16C cycling populations (*thin arrows).

Figure 4 depicts the 24 h (t24), 48 h (t48), and 72 h (t72) cell cycle data for untreated and 1 μM cabazitaxel-treated SKOV3 and TOV-112D cells. As compared to untreated SKOV3 cells at 24 h, cabazitaxel-treated SKOV3 cells show accumulation at a 4C G2-phase indicated by intense cyclin A2 signal (e.g., thin arrow). There is also a substantial M-phase arrest as marked by intense PHH3 signal (e.g., thick arrow). The presence of 4C G1 SKOV3 cells as the predominant interphase population at 48 h and at 72 h (double arrows), the predominance of 4C M cells at 48 h (thick double arrows), and the reduction of 4C M cells at 72 h suggests that the cabazitaxel-induced M-phase arrest is transient. SKOV3 cells resolve the arrest by entering a cell cycle without mitosis or cytokinesis (4C → 8C or 8C → 16C). Cytometry analyses of TOV-112D cells shows similar outcomes, but TOV-112D cells move efficiently through higher order (8C or 16C) cell cycles (*thin arrows).

Radiation (2 Gy) when added to 1 μM cabazitaxel treatment did not change the cabazitaxel effects reflected by cyclin A2 or PHH3 in SKOV3 cells. However, the presence of sub G1 events indicating fragmented DNA (and lethal cell events) appears enhanced in the irradiated SKOV3 cells. By comparison, irradiated TOV-112D cells showed 4C G2 arrest at 24, 48, and 72 h, a profound 4C G1 arrest at 48 h and at 72 h, and a mitotic arrest at 24 and 48 h that is linked to lower PHH3 levels. The latter finding indicates that the two cell lines are responding differently at the molecular level, and further partitioning of the G2/M-phase could detail such a molecular “fingerprint.” Taken together, high-content flow cytometry suggests cabazitaxel administered with or without radiation results in mitotic arrest followed by an escape to a higher order DNA content cell cycle that may or may not be further damaged by radiation.

**Figure 5:**
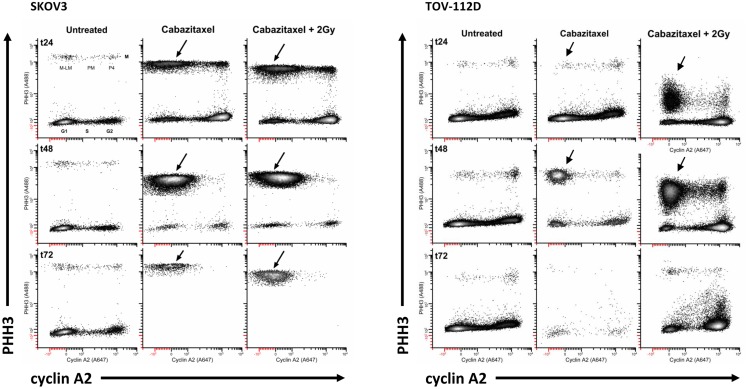
**Mitotic state data are shown for SKOV and TOV-112D cells**. A 6-h cabazitaxel (1 μM) exposure was studied; drug-free medium was exchanged at the 6-h time point. 24 h (t24), 48 h (t48), and 72 h (t72) after initial cabazitaxel treatment is indicated for each row of cytometry data. Plots are PHH3 (phospho-S10-histone H3) versus cyclin A2. Mitotic cells have elevated PHH3 and three mitotic states can be quantified (P4, PM, M-LM). These are named as previously described (42). Arrows point out M-LM.

A typical mitotic phenotype for microtubule poisons is arrest at metaphase (cyclin A2 levels are background) or prometaphase (chromosomes are not attached to a spindle). An expected cabazitaxel-induced phenotype is residence of cells in a PHH3 high/cyclin A2 low level state (M-LM state). Figure 5 shows high-content flow cytometry plots of PHH3 level versus cyclin A2 level at 24 h (t24), 48 h (t48), and 72 h (t72). Effects of 1 μM cabazitaxel alone and radiation (2 Gy)-cabazitaxel (1 μM) are contrasted with untreated SKOV3 and TOV-112D cells. For both cell lines, mitotic arrest occurred in a M-LM state (arrows). Other than a large difference in the number of cells residing in M-LM state, both cells lines show a cabazitaxel-induced M-phase arrest consistent with activating the spindle checkpoint that is unaffected by radiation.

## Discussion

Cell cycle phase impact upon cell radiosensitivity has been studied extensively (5, 32), with attempts to enhance radiosensitivity described by pharmacological manipulation of the cell cycle (33). In this work, the new taxane cabazitaxel demonstrated concentration-dependent cytotoxicity and radiosensitization. While subtle differences in cabazitaxel sensitivity and radiosensitization were detected, cabazitaxel did show lethal effects in cells known to evade taxane cytotoxicity. Among four tested schedules, cabazitaxel added 24 h before radiation enhanced the lethal effects most. Cytometry confirmed that cabazitaxel-treated cells accumulated in a radiosensitive G2/M 4C DNA complement. Cytometry indicated that surviving cabazitaxel-treated cells resolved arrest by release from the mitotic spindle checkpoint response and entry into subsequent 4C or 8C cell cycles, in accordance with prior taxane studies (34, 35).

A precise mechanism by which an augmented G2/M-phase cell cycle arrest sensitizes cells to radiation is not well understood. An enhanced susceptibility to radiation-induced double-strand breaks in tightly packaged DNA could partially explain the radiosensitivity we observed; however, such an explanation cannot account for the cytotoxic effects of cabazitaxel alone where mitotic checkpoint responses are likely to be active and protracted response activation may be lethal as well. Attempted G2/M to G1 traversal in the setting of unrepaired DNA damage is a toxic event, likely due to activation of mitotic cell death responses and possible loss of vital genetic material in cell progeny through unrepaired DNA double-strand breaks (36). It also may be that radiation-induced, closely situated single-strand breaks are converted to toxic double-strand breaks over time due to drift of damaged DNA break ends.

We put forward that stalling of G2/M-phase traversal by cabazitaxel explains supraadditive interactions when cabazitaxel is administered 18–24 h before ionizing radiation. This arrest mechanism is additionally supported by the resolution of the G2/M-phase block through either a mitotic spindle checkpoint response or endoreduplication. Administering radiation and cabazitaxel treatment together appears additive, as the cell cycle perturbing effects of cabazitaxel are not apparent for several hours later. Giving radiation and chasing it 24 h later with cabazitaxel results in mixed response. The cumulative data suggests that in a radiation-cabazitaxel therapeutic strategy, the optimal sequence is cabazitaxel first and radiation preferably 24 h later. Pharmacokinetic and pharmacodynamic experiments in mice have been published to guide human clinical trials (37). Phase I trials of radiation-cabazitaxel are needed to clinically confirm or refute radiation-cabazitaxel sequencing. Other mechanisms of cell cycle manipulation may be at play in our findings – undocumented coordination of or disruption of intracellular signaling pathways, unrecorded molecular events related to apoptosis, or possibly unobserved differential paclitaxel and cabazitaxel affinity for transmembrane P-glycoprotein transporters.

Paclitaxel is utilized commonly in the clinical management of women with ovarian cancer (38–40). It is apparent from clinical trials that there is a differential response to therapy, with residual chemorefractory disease harboring no clear-cut molecular signal to explain this difference. A novel taxane such as cabazitaxel may have desirable cancer cell cytotoxic effects on its own and effects on the cell cycle that sensitize cells to radiation. It may be envisioned that a second-line radiochemotherapy strategy could emerge for the management of incompletely responding persistent or recurrent ovarian cancers. Here, radiosurgery (41) could target and ablate sites of chemorefractory ovarian cancer while cabazitaxel provides radiosensitization and independent cytotoxicity. The combination of radiosurgery and concomitant chemotherapy (i.e., gemcitabine and carboplatin) is already being studied in a phase I clinical trial (listed at http://clinicaltrials.gov/ as NCT01652794). Therefore, we believe that the enhanced radiosensitivity that taxanes such as cabazitaxel provides a rationale for testing of cabazitaxel and radiation in short order.

## Conflict of Interest Statement

Sanofi-Aventis provided a research grant to the Department of Radiation Oncology and The Case Western Reserve University School of Medicine whose funds supported some research activities related to this manuscript.
